# Rapid climate action is needed: comparing heat vs. COVID-19-related mortality

**DOI:** 10.1038/s41598-024-82788-8

**Published:** 2025-01-06

**Authors:** Fulden Batibeniz, Sonia I. Seneviratne, Srinidhi Jha, Andreia Ribeiro, Laura Suarez Gutierrez, Christoph C. Raible, Avni Malhotra, Ben Armstrong, Michelle L. Bell, Eric Lavigne, Antonio Gasparrini, Yuming Guo, Masahiro Hashizume, Pierre Masselot, Susana Pereira da Silva, Dominic Royé, Francesco Sera, Shilu Tong, Aleš Urban, Ana M. Vicedo-Cabrera

**Affiliations:** 1 Department of Environmental Systems Science, Institute for Atmospheric and Climate Science, ETH Zurich, Zurich, Switzerland; 2 Oeschger Centre for Climate Change Research (OCCR), University of Bern, Bern, Switzerland; 3 Climate and Environmental Physics, Physics Institute, University of Bern, Bern, Switzerland; 4 Institut Pierre-Simon Laplace, CNRS, Paris, France; 5 Biological Sciences Division, Pacific Northwest National Laboratory, Richland, WA USA; 6 Department of Public Health Environments and Society, London School of Hygiene and Tropical Medicine, London, UK; 7 School of the Environment, Yale University, New Haven, CT USA; 8 School of Health Policy and Management, College of Health Sciences, Korea University, Seoul, 02841 Republic of Korea; 9 Faculty of Medicine, School of Epidemiology and Public Health, University of Ottawa, Ottawa, Canada; 10 Environmental Health Science and Research Bureau, Health Canada, Ottawa, Canada; 11 Environment and Health Modelling (EHM) Lab, Department of Public Health Environments and Society, London School of Hygiene and Tropical Medicine, London, UK; 12 Department of Epidemiology and Preventive Medicine, School of Public Health and Preventive Medicine, Monash University, Melbourne, Australia; 13 Climate, Air Quality Research Unit, School of Public Health and Preventive Medicine, Monash University, Melbourne, Australia; 14 Department of Global Health Policy, Graduate School of Medicine, The University of Tokyo, Tokyo, Japan; 15 Department of Epidemiology, Instituto Nacional de Saúde Dr. Ricardo Jorge, Lisbon, Portugal; 16 Climate Research Foundation (FIC), Madrid, Spain; 17 CIBERESP, Madrid, Spain; 18 Department of Statistics, Computer Science and Applications “G. Parenti”, University of Florence, Florence, Italy; 19 Chinese Center for Disease Control and Prevention, National Institute of Environmental Health, Beijing, China; 20 School of Public Health and Social Work, Queensland University of Technology, Brisbane, Australia; 21 Institute of Atmospheric Physics, Czech Academy of Sciences, Prague, Czech Republic; 22 Faculty of Environmental Sciences, Czech University of Life Sciences, Prague, Czech Republic; 23 Institute of Social and Preventive Medicine (ISPM), University of Bern, Bern, Switzerland

**Keywords:** Climate sciences, Climate change

## Abstract

The impacts of climate change on human health are often underestimated or perceived to be in a distant future. Here, we present the projected impacts of climate change in the context of COVID-19, a recent human health catastrophe. We compared projected heat mortality with COVID-19 deaths in 38 cities worldwide and found that in half of these cities, heat-related deaths could exceed annual COVID-19 deaths in less than ten years (at + 3.0 °C increase in global warming relative to preindustrial). In seven of these cities, heat mortality could exceed COVID-19 deaths in less than five years. Our results underscore the crucial need for climate action and for the integration of climate change into public health discourse and policy.

## Introduction

Climate change poses a catastrophic threat to humanity. The increasing frequency of extreme temperature events, along with overall warming^1,2^, has resulted in a significant rise in heat-related health burden^3,4^ which is projected to persist or increase under warmer conditions^3,5,6^. The recent COVID-19 pandemic also represented an unprecedented public health catastrophe with a substantial mortality burden worldwide. Here, we estimate the current and future impacts of climate change on human mortality by using the COVID-19 pandemic as a benchmark.

**Fig. 1:**
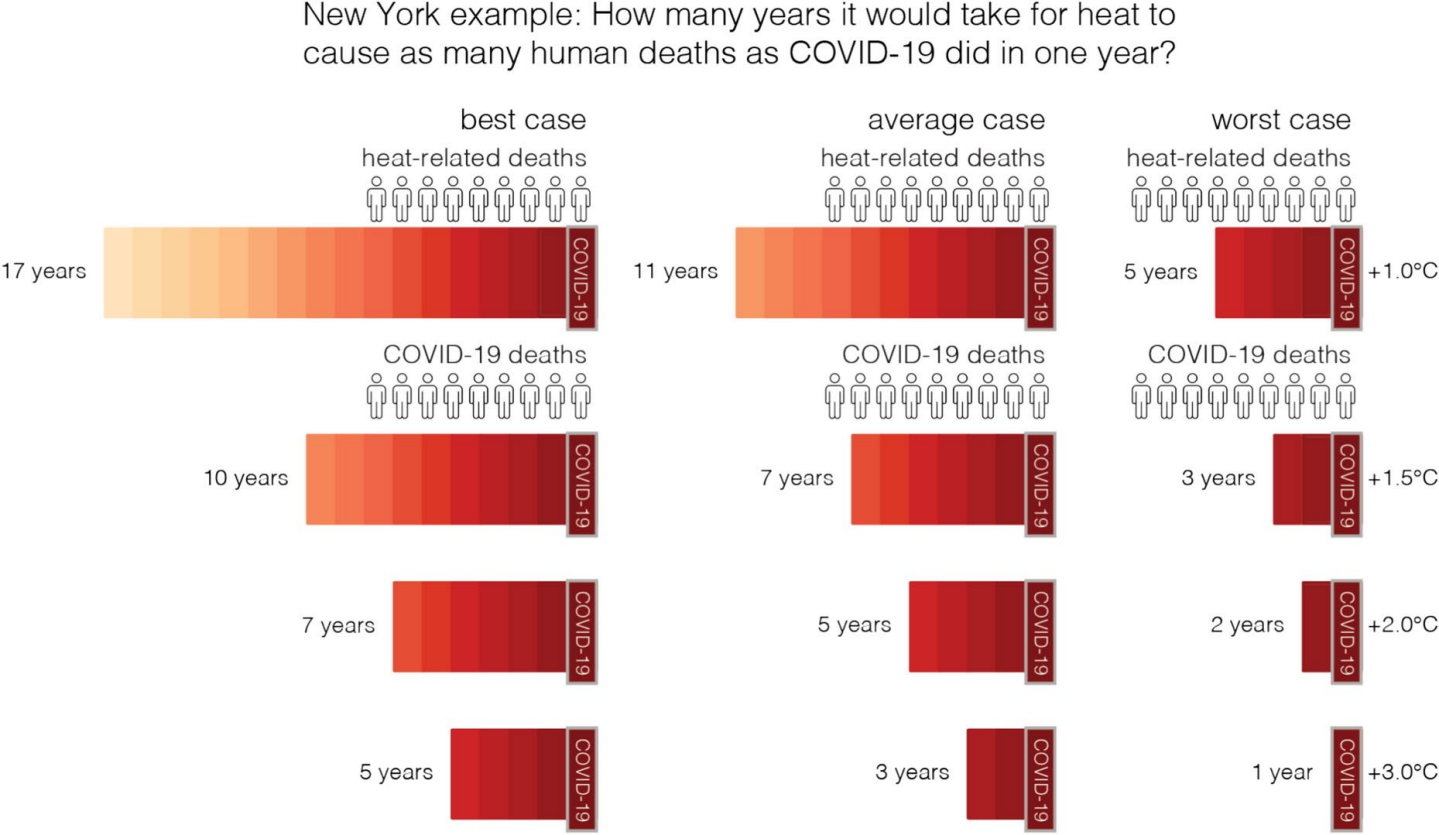
Comparison between heat- and COVID-19-related mortality for New York. Each bar illustrates the number of years it may take for cumulative heat-related deaths to equal 1 year of COVID-19 pandemic mortality. We present the best case (lower bound), average case (median) and worst case (upper bound) at GWLs of + 1 °C, + 1.5 °C, + 2 °C and + 3 °C. The median, lower and upper bounds are derived using 1000 Monte Carlo simulations (see “Materials and methods” section).

We calculated the number of years it would take for heat-related deaths to equal a single year’s worth of COVID-19 deaths in 38 highly populated cities across 34 countries spanning six continents. Using well-established epidemiological methods^4,5^, we combined location-specific temperature-mortality relationship for each city with bias-corrected temperature simulations from 31 global climate models from the sixth phase of the Coupled Model Intercomparison Project (CMIP6) to project heat-related mortality on different global warming levels (GWLs of + 1 °C, + 1.5 °C, + 2 °C and + 3 °C). For each GWL, we compared the heat-related mortality fractions (%, i.e., percentage of deaths due to heat over total mortality) in each city to the national average mortality fraction for COVID-19 (2020–2021) using data from the Center for Systems Science and Engineering (CSSE) at Johns Hopkins University^7,8^ and World Development Indicators^9^. As an illustration of our approach, if 1 out of 100 deaths in a year were heat-related (1%), and 20% were due to COVID-19, our key metric, the number of years until heat-related deaths equate one year of COVID-19 deaths, would be 20 years. We present the median of the 31 CMIP6 models as the average case and the upper bound as the worst case and the lower bound as the best case within the 95% confidence intervals (Fig. 1, Supplementary Table 1). Full spread of the models is shown in Fig. 3.

**Fig. 2:**
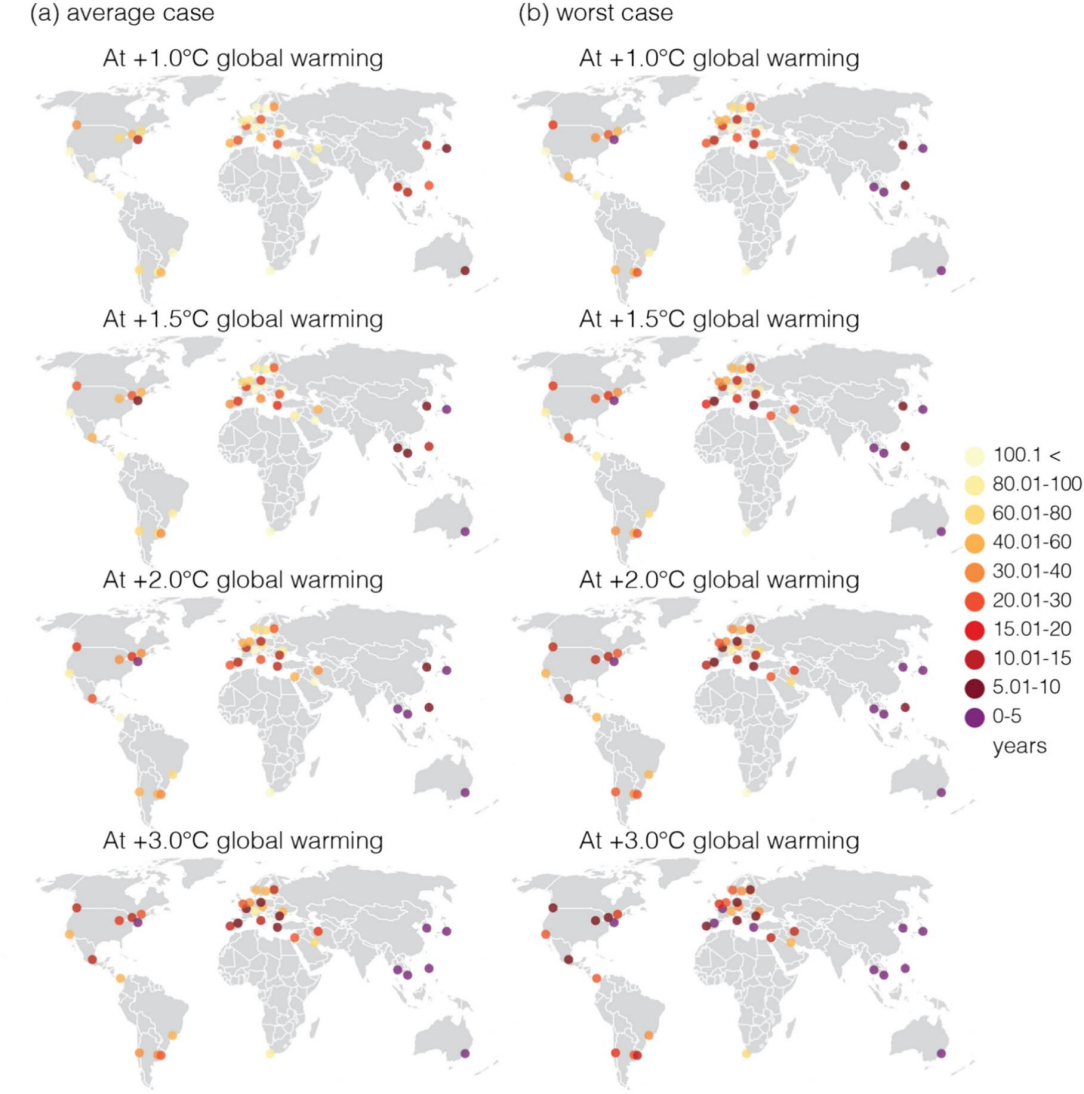
The number of years needed for heat-related mortality to equate 1-year of COVID-19-related mortality. (**a**) Average case: the medians of the models, (**b**) worst case: the upper range of models, within the 95% confidence intervals.

**Fig. 3:**
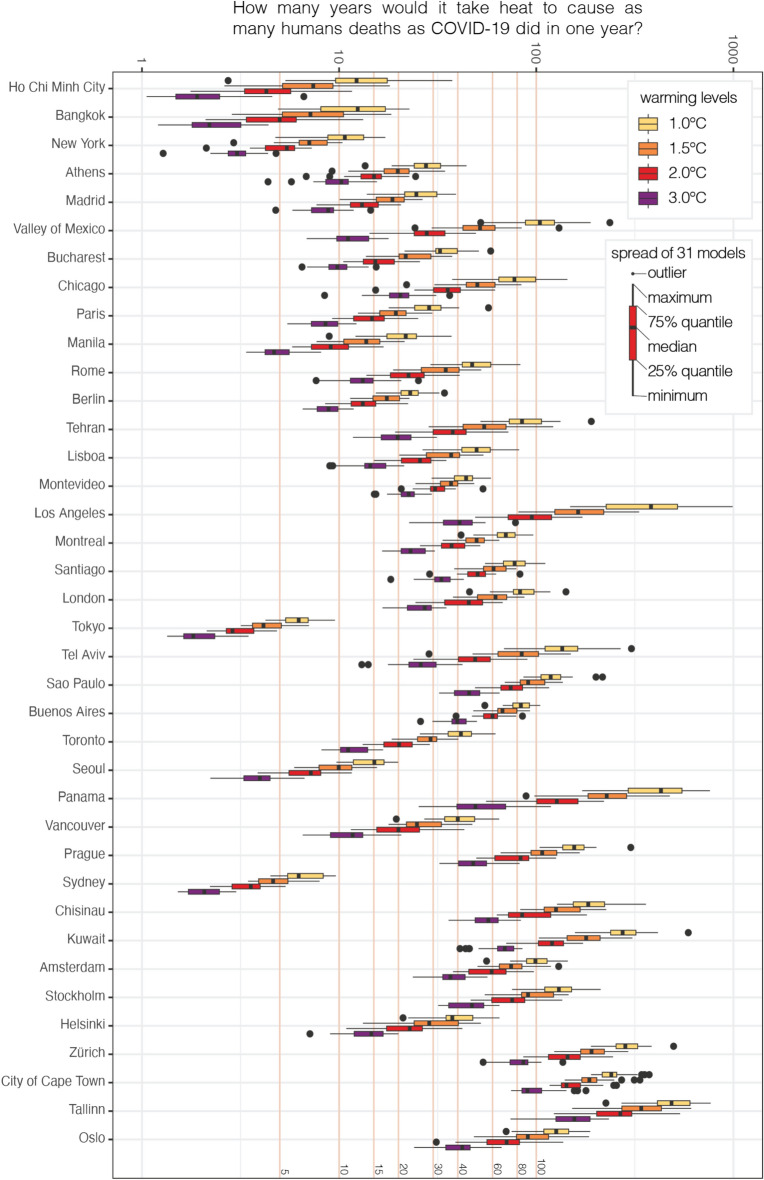
The number of years needed for heat-related mortality to equate 1-year of COVID-19-related mortality for all the models (the ratio between COVID-19 mortality and heat-related mortality) at GWLs of + 1 °C, + 1.5 °C, + 2 °C and + 3 °C. Box plots display the distribution of this ratio across 31 models (in years). The x-axis is displayed logarithmically for clarity. Orange lines serve as a reference to the scale used in Fig. 2a,b.

We found that heat-related mortality in cities can reach COVID-19 mortality levels faster under higher global warming levels, assuming current vulnerability and socioeconomic structures remain unchanged. This holds true for cities with both high and low COVID-19 mortality rates. We identified New York as one of the cities that is highly vulnerable to both COVID-19 and climate change (Fig. 1). In the average case (best case), we estimated that heat-related deaths in New York could equal COVID-19 deaths within 11 years (17 years) at + 1.0 °C and within 3 years (5 years) at + 3.0 °C. In the worst case, these levels could be reached even sooner, within 5 years at + 1.0 °C and within 1 year at + 3.0 °C. At the current GWL (+ 1.0 °C), two cities (Sydney and Tokyo) are already at a point where heat-related deaths could equal COVID-19 deaths within 10 years (Figs. 2, 3, Supplementary Table 1). In the worst case, this estimate for the current GWL extends to 7 cities (Sydney, Tokyo, New York, Bangkok, Ho Chi Minh City, Manila, and Seoul). Note that cities in Asia and Australia have lower COVID-19 burdens compared to other cities in the study. As GWLs increase, the number of cities expected to experience heat-related deaths equal to COVID-19 deaths within 10 years increases: 6 cities (15.8%, e.g., Manila) at + 1.5 °C, 7 cities (18.4%, e.g., Berlin, Madrid, Paris, Athens, Bucharest) at + 2.0 °C, and 12 cities (31.6%, e.g., Valley of Mexico, Rome, Lisbon, Helsinki, Vancouver, and Toronto) at + 3.0 °C. In the worst case, the numbers are even higher: 9 cities (23.7%, e.g., Madrid, Athens) at + 1.5 °C, 11 cities (29.0%, e.g., Berlin, Paris) at + 2.0 °C, and 19 cities (50.0%, e.g., Chicago, Bucharest, Valley of Mexico, Rome, Lisbon, Helsinki, Vancouver, and Toronto) at + 3.0 °C. North American and European cities, particularly those in the Mediterranean and Central Europe, show a sharp increase in heat-related deaths with each increment in global warming, indicating a shorter duration (less than 5 years for some cities at + 3.0 °C) to reach COVID-19 mortality. These regions are considered as hotspots for climate change^1,2,10^, in particular for heat-related mortality due to their increased vulnerability and exposure.

Our findings serve as a reminder that every bit of warming is significant for human lives. The year 2023 further exemplified this, marked by unprecedented global climate shifts and heat extremes^11^. For several months, the mean global temperature anomaly exceeded 1.5 °C for the first time in recorded history. This rise in temperature, accompanied by major extreme climate events led to an increase in heat-related deaths. For example, a record number of 334 people died from heat in Texas in 2023^12^. Human-induced climate change significantly contributed to the heat-related mortality in Switzerland also during the summer of 2022, with 60% of the estimated 623 heat-related deaths could have potentially been avoided in the absence of human-induced climate change^13^. Our study suggests that climate change will exacerbate heat-related mortality. The extent of the increase may depend on factors such as local climate, healthcare infrastructure, and mitigation measures. Beyond the immediate toll on human lives, the impacts of climate change, including heat-related mortality and extreme heat events, have significant economic implications. In a previous study, we estimated that global GDP would be 3.2% lower at + 1.5 °C and 10.0% lower at a + 3.0 °C increase in global warming compared to preindustrial levels^14^. These economic losses at + 3.0 °C exceed the GDP reduction caused by both the COVID-19 pandemic and the global financial crisis in 2009^14^, with nearly half of these damages attributed to extreme heat^14^.

While our study provides a useful benchmark to emphasize the urgency of climate change impacts on human health, there are caveats with our approach. Firstly, our approach is conservative as we did not consider population changes and demographic factors. Projected population increases and an aging demographic could potentially exacerbate the severity of heat-related mortality^15^. Secondly, we did not account for changes in heat vulnerability (i.e., adaptation), which could have resulted in a lower mortality burden. Future analyses could consider accounting for the complexity and uncertainties steaming from modelling adaptation^16^. However, it is important to note that adaptation cannot be compressively included as there is no consensus on the best adaptation measures for heat-related mortality. Thirdly, the heat mortality database that we used (see “Materials and methods” section) does not cover the totality of all population for most of the countries included. Therefore, we restricted the analysis to one city per country (or a sample of cities in the U.S.), which may not reflect the country-wide pattern. Fourthly, we used the average COVID-19 mortality in a country as the representative burden for each city since city-level data were inconsistently available. Since COVID-19 was substantially higher in main cities compared to rural or smaller locations^17^, potential differences in COVID-19 mortality within countries and underreporting of cases also need to be considered when drawing inferences from our results. Finally, we did not consider the dependency between COVID-19 and heat-related mortality, which could lead to a higher mortality burden^18^. A recent study found that the combined impact of heatwaves and COVID-19 led to an increase in excess deaths per 100,000 population in some regions of England between 2020 and 2022, doubling the rates of the previous decade^18^.

In conclusion, our study reveals that the effects of increasing temperatures on human lives are comparable to and may exceed mortality from COVID-19. Effective measures to mitigate climate change are crucial for protecting global human health and well-being in the present and future. By comparing heat-related mortality to COVID-19 mortality, we provide insight into the urgency of climate change through the lens of a recent and catastrophic global pandemic. Moreover, it is important to remember that the direct heat exposure is just one factor contributing to climate-related public health impacts. Therefore, our climate-related mortality impact estimate is conservative. The consequences of climate change are not as distant as they may seem, and the threat to human lives is comparable to the profound global impact of the COVID-19 pandemic.

## Materials and methods

### Observed temperature and mortality data: the multi-country multi-city (MCC) collaborative research network database

We obtained recorded daily temperature and mortality data for 38 cities from the MCC Collaborative Research Network database, the largest weather and health data consortium to date. The data include daily mortality counts due to any cause or only non-external causes, and the daily average temperature (°C) from local weather stations (Supplementary Table 2). The length of the available data varies by location. Supplementary Table 2 presents descriptive statistics for each city.

### COVID-19 mortality data

We used the COVID-19 Data Repository by the Center for Systems Science and Engineering (CSSE) at Johns Hopkins University. The repository is an online interactive dashboard that tracks reported cases of COVID-19 in real time^7,8^. The global data provided by CSSE is daily and at a national level. Detailed information regarding the data and its sources are available under: https://github.com/CSSEGISandData/COVID-19. We retrieved crude death rate (per 1000 people) and total population of each country from the World Development Indicators website https://databank.worldbank.org/source/world-development-indicators/preview/on and calculated the total mortality for 2020 and 2021 (Supplementary Table 3).

To compare mortality due to COVID-19 with heat-related excess mortality, we used the ratio between the total COVID-19-related deaths and the total country mortality for the years 2020 and 2021. In 2020 and 2021, we selected the year with a higher mortality burden for each city, considering that some cities faced a greater impact from the omicron strain of COVID-19 in late 2021 (Supplementary Figure 2, Supplementary Table 3). Since city-level COVID-19 data was only available for certain cities and from varied sources, we used country-level mortality and overall mortality figures to estimate the percentage of COVID-19-related deaths. In regions where state-level data were available, such as the U.S. and Canada, we used these data for corresponding cities. This allowed us to make meaningful comparisons between COVID-19 mortality and heat-related mortality, despite the lack of city-level COVID-19 data.

### Climate dataset and study period definition across warming levels

We used CMIP6 simulations from 31 distinct climate models (as shown in Supplementary Table 4). Our analysis covered four Global Warming Levels (GWLs) during present conditions (+ 1 °C), and three higher GWLs (+ 1.5 °C, + 2 °C, + 3 °C) relative to the preindustrial period 1850–1900. These GWLs span 20-year periods and are defined separately for each model to account for variations in climate sensitivity and internal variability. We performed our analysis using GWLs to align with the IPCC AR6 context, inform policy makers and refer to the targets of the Paris Agreement. For more information on the method used to calculate GWLs, we refer Batibeniz et al.^1^ and Seneviratne et al.^2^.

We used the Shared Socioeconomic Pathway (SSP), SSP5-8.5, which represents a scenario characterized by high-mitigation and low-adaptation challenges, resulting in a radiative forcing of 8.5 Wm^2^ by the year 2100^19,20^. Since our findings are presented across GWLs, the choice of scenario is not expected to have a significant impact on our results, consistent with previous research^21–23^.

For our analysis, we used daily mean temperature data for 38 cities obtained from each model using the nearest-neighbour method based on latitude and longitude. We performed bias correction using local weather station data from the MCC Collaborative Research Network database. An adopted quantile mapping method provides robust climate model-based scenarios for stations. The approach involves climate model bias correction and spatial transfer of bias-corrected model data to represent the characteristics of the station. The method is validated, and results show promising performance for the mean temperature. This process follows the method outlined in previous studies^24^.

### Estimation of exposure–response relationships of temperature and mortality

The analysis of the relationship between heat and mortality in each location during the period of 1991–2019 was conducted through a two-stage time-series approach, which is a common method^4,5,25^.

In the first stage, we used quasi-Poisson regression time series analyses with distributed lag nonlinear models (DLNM) to estimate the temperature-mortality association for each location^26^. Mean temperature was chosen as the exposure variable^27–30^, and the model specification and parameterization were based on previous studies^27,31,32^. To account for long-term trends and seasonal patterns, we incorporated a natural cubic spline representing time, utilizing eight degrees of freedom for each year, in addition to an indicator term for the day of the week. The temperature-mortality curve was modelled with a natural-spline with three internal knots placed at the 10th, 75th, and 90th percentile of the location-specific observational temperature distributions^33^. A natural cubic spline with three internal knots equally distributed up to 21 days was applied to capture the lagged response of mortality. The exposure–response function was then reduced into a one-dimensional cumulative exposure–response function, which expresses the location-specific relative risk of mortality as a function of local daily mean temperature.

In the second stage, location-specific coefficients from the first stage were pooled in a multivariate meta-regression model to make full use of the hierarchical structure of the data^34^. We included a set of meta-predictors to capture part of the heterogeneity across locations, such as indicators for region, climate classification^35^, country-level gross domestic product per capita, and location-specific average and range of temperature. Best linear unbiased predictions (BLUPs) were derived to represent improved location-specific estimates, especially for locations with a short time series or low mortality counts. The BLUPs were then log-linearly extrapolated to cover the additional range of temperature occurring in the GWLs. The analysis was conducted using the R software environment, with the packages dlnm^36^ and mixmeta^34^.

### Projection of the heat impact on mortality

We projected the heat-related mortality impacts by combining the exposure–response curve with the temperature projections from the 31 CMIP6 global climate models (GCMs) (Supplementary Figure 1). We assumed no adaptation or population changes. We followed the method described in Vicedo-Cabrera et al. (2019) for climate change projections^25^. Using the relative risk [using minimum mortality temperature (MMT) as reference] corresponding to each day’s temperature, we computed the attributable deaths due to temperature. Heat-related deaths were analysed for each city, global warming level and model. The total heat-related mortality attributed was computed by summing the subsets corresponding to days with temperatures higher than the minimum mortality temperature for 20-year global warming levels. The heat-mortality fraction was then computed as the percentage of heat-related deaths over the total mortality. 1000 Monte Carlo simulations were used to obtain empirical confidence intervals (eCIs) that quantified the uncertainty in both the estimation of the exposure-lag-response relationships and climate projections across GCMs (Supplementary Fig. 1, Supplementary Table 5).

### Calculation of the comparison between COVID-19 and heat

Given the significant but short-term impact of the COVID-19 pandemic, we selected COVID-19 as a benchmark for our analysis. This decision enables us to highlight the urgency of climate change by drawing parallels between the mortality associated with heat and that attributed to COVID-19. The COVID-19 pandemic was an unprecedented event responsible for a substantial mortality burden. However, a pandemic with the same or greater intensity as the COVID-19 outbreak is only expected to occur once every 209 years^37^. Although the risk of dying due to heat could be lower than due to COVID-19, heat-related deaths happen every year.

To make this comparison possible, we developed a method to represent heat-related mortality in terms of COVID-19 mortality. The metric gives us the number of years that the total amount of heat deaths would be equal to 1 year of COVID-19 deaths either in 2020 or 2021. In a hypothetical example where there are 100 all-cause deaths per year, the fraction of heat mortality per year is 1% (1 person), and COVID-19 is 20% (20 persons) for 1 year. Therefore, the ratio of the mortality fraction of COVID-19 (20% for 1 year) to heat (average 1% per year) would be 20 years. Thus, the cumulative number of heat-related deaths over 20 years would be equivalent to one year of COVID-19 deaths. This means that one year of COVID-19 deaths would correspond to 20 years of heat-related deaths, enabling us to illustrate the urgency of climate change in terms of COVID-19. The reason we use fractions instead of the actual number of deaths is because COVID-19 data are only available at the country level, and total mortality at city level.

## Supplementary Information

Supplementary Information.

## Data Availability

Daily mean temperature data were retrieved from CMIP6 repository of ETH Zurich (10.5281/ZENODO.3734128)^38^. Note that CMIP6 model outputs are also available from different Earth System Grid Federation (ESGF) data nodes. Mortality data were collected within the MCC Collaborative Research Network under a data sharing agreement and cannot be made publicly available. Individual datasets may be accessible upon request to the corresponding MCC partners.

## Code availability

All codes necessary to reproduce the analysis presented in this study are available in the following GitHub repository: https://github.com/fuldenica/COVID-19-vs.-Heat-related-mortality/tree/main?tab=readme-ov-file. This repository contains the data processing scripts, statistical analysis code, and visualization scripts used in our research.
