# Supplementary material for: Rapid climate action is needed: comparing heat vs. COVID-19-related mortality
Source: Sci Rep. 2025 Jan 6;15:1002. doi: 10.1038/s41598-024-82788-8 (PMC11704295; doi:10.1038/s41598-024-82788-8)
Supplement: Supplementary file 1 — Supplementary Information. [file 41598_2024_82788_MOESM1_ESM.docx]

**Rapid Climate Action is Needed: Comparing Heat vs. COVID-19-related Mortality**

Fulden Batibeniz^1,2,3*^, Sonia I. Seneviratne^1*^, Srinidhi Jha^1^, Andreia Ribeiro^1^, Laura Suarez Gutierrez^1,4^, Christoph C. Raible^2,3^, Avni Malhotra^5^, Ben Armstrong^6^, Michelle L. Bell^7,8^, Eric Lavigne^9,10^, Antonio Gasparrini^11^, Yuming Guo^12,13^, Masahiro Hashizume^14^, Pierre Masselot^11^, Susana Pereira da Silva^15^, Dominic Royé^16,17^, Francesco Sera^18^, Shilu Tong^19,20^, Aleš Urban^21,22^, Multi-Country Multi-City Collaborative Research Network^§^, Ana M. Vicedo-Cabrera^2,23*^

^1^Institute for Atmospheric and Climate Science, Department of Environmental Systems Science, ETH Zurich, Zurich, Switzerland

^2^Oeschger Centre for Climate Change Research (OCCR), University of Bern, Bern, Switzerland

^3^Climate and Environmental Physics, Physics Institute, University of Bern, Bern, Switzerland

^4^Institut Pierre-Simon Laplace, CNRS, Paris, France

^5^Biological Sciences Division, Pacific Northwest National Laboratory, Richland, WA, USA

^6^Department of Public Health Environments and Society, London School of Hygiene & Tropical Medicine, London, United Kingdom

^7^School of the Environment, Yale University, New Haven CT, USA

^8^School of Health Policy and Management, College of Health Sciences, Korea University, Seoul 02841, Republic of Korea

^9^School of Epidemiology & Public Health, Faculty of Medicine, University of Ottawa, Ottawa, Canada

^10^Environmental Health Science & Research Bureau, Health Canada, Ottawa, Canada

^11^Environment & Health Modelling (EHM) Lab, Department of Public Health Environments and Society, London School of Hygiene & Tropical Medicine, London, UK

^12^Department of Epidemiology and Preventive Medicine, School of Public Health and Preventive Medicine, Monash University, Melbourne, Australia

^13^Climate, Air Quality Research Unit, School of Public Health and Preventive Medicine, Monash University, Melbourne, Australia

^14^Department of Global Health Policy, Graduate School of Medicine, The University of Tokyo, Tokyo, Japan

^15^Department of Epidemiology, Instituto Nacional de Saúde Dr. Ricardo Jorge, Lisbon, Portugal

^16^Climate Research Foundation (FIC), Madrid, Spain

^17^CIBERESP, Madrid. Spain

^18^Department of Statistics, Computer Science and Applications "G. Parenti", University of Florence, Florence, Italy

^19^National Institute of Environmental Health, Chinese Center for Disease Control and Prevention, Beijing, China

^20^School of Public Health and Social Work, Queensland University of Technology, Brisbane, Australia

^21^Institute of Atmospheric Physics, Czech Academy of Sciences, Prague, Czech Republic

^22^Faculty of Environmental Sciences, Czech University of Life Sciences, Prague, Czech Republic

^23^Institute of Social and Preventive Medicine (ISPM), University of Bern, Bern, Switzerland

^§^ Group of authors provided in supplementary file.

* Corresponding authors: Fulden Batibeniz ([fulden.batibeniz@env.ethz.ch](mailto:fulden.batibeniz@env.ethz.ch)), Sonia I. Seneviratne (sonia.seneviratne@ethz.ch), Ana M. Vicedo-Cabrera ([anamaria.vicedo@unibe.ch](mailto:anamaria.vicedo@unibe.ch))

# **Supplementary information**

**Multi-Country Multi-City Collaborative Research Network:**

Rosana Abrutzky^1^, Micheline de Sousa Zanotti Stagliorio Coelho^2^, Paulo Hilario Nascimento Saldiva^3^, Patricia Matus Correa^4^, Nicolás Valdés Ortega^5^, Jan Kyselý^6,7^, Hans Orru^8^, Ene Indermitte^8^, Jouni J. K. Jaakkola^9,10^, Niilo Ryti^9,10^, Mathilde Pascal^11^, Alexandra Schneider^12^, Veronika Huber^13,12^, Klea Katsouyanni^14,15^, Antonis Analitis^14^, Fatemeh Mayvaneh^16^, Hematollah Roradeh^17^, Raanan Raz^18^, Paola Michelozzi^19^, Francesca de'Donato^19^, Masahiro Hashizume^20^, Yoonhee Kim^21^, Barrak Alahmad^22^, Magali Hurtado Diaz^23^, Eunice Elizabeth Félix Arellano^23^, Ala Overcenco^24^, Danny Houthuijs^25^, Caroline Ameling^25^, Shilpa Rao^26^, Xerxes Seposo^27,28^, Paul Lester Carlos Chua^29^, Joana Madureira^30,31,32^, Iulian-Horia Holobaca^33^, Noah Scovronick^34^, Fiorella Acquaotta^35^, Ho Kim^36^, Whanhee Lee^37^, Aurelio Tobias^38^, Carmen Íñiguez^39,40^, Bertil Forsberg^41^, Martina S. Ragettli^42,43^, Shanshan Li^44,45^, Valentina Colistro^46^, Antonella Zanobetti^47^, Joel Schwartz^47^, Tran Ngoc Dang^48^, Do Van Dung^48^, Antonio Gasparrini^49^, Michelle L. Bell^50^, Yuming Guo^44,45^, Yasushi Honda^51^, Aleš Urban^6,7^, Ana Maria Vicedo-Cabrera^52,53^, Pierre Masselot^49^, Ben Armstrong^54^, Eric Lavigne^55,56^, Susana Pereira da Silva^57^, Dominic Royé^58,40^, Francesco Sera^59^, Shilu Tong^60,61^

^1^Universidad de Buenos Aires, Facultad de Ciencias Sociales, Instituto de Investigaciones Gino Germani

^2^Department of Pathology, Faculty of Medicine, University of São Paulo, São Paulo, Brazil

^3^INSPER, São Paulo, Brazil

^4^Department of Public Health, Universidad de los Andes, Santiago, Chile

^5^Centro Interdisciplinario de Cambio Global, Pontificia, Universidad Católica de Chile, Santiago, Chile

^6^Institute of Atmospheric Physics, Czech Academy of Sciences, Prague, Czech Republic

^7^Faculty of Environmental Sciences, Czech University of Life Sciences, Prague, Czech Republic

^8^Department of Family Medicine and Public Health, University of Tartu, Tartu, Estonia

^9^Center for Environmental and Respiratory Health Research (CERH), University of Oulu, Oulu, Finland

^10^Medical Research Center Oulu (MRC Oulu), Oulu University Hospital and University of Oulu, Oulu, Finland

^11^Santé Publique France, Department of Environmental and Occupational Health, French National Public Health Agency, Saint Maurice, France

^12^Institute of Epidemiology, Helmholtz Zentrum München – German Research Center for Environmental Health (GmbH), Neuherberg, Germany

^13^IBE-Chair of Epidemiology, LMU Munich, Munich, Germany

^14^Department of Hygiene, Epidemiology and Medical Statistics, National and Kapodistrian University of Athens, Greece

^15^Environmental Research Group, School of Public Health, Imperial College, London, UK

^16^University of Münster, Institute of Landscape Ecology, Climatology Research Group, Münster, Germany

^17^Geography and Urban Planning Department, University of Mazandaran, Babolsar, Iran

^18^Braun School of Public Health and Community Medicine, The Hebrew University of Jerusalem, Israel

^19^Department of Epidemiology, Lazio Regional Health Service, Rome, Italy

^20^Department of Global Health Policy, Graduate School of Medicine, The University of Tokyo, Tokyo, Japan

^21^Department of Global Environmental Health, Graduate School of Medicine, University of Tokyo, Tokyo, Japan

^22^Department of Environmental Health, Harvard T.H. Chan School of Public Health, Harvard University, Boston, MA, USA

^23^Department of Environmental Health, National Institute of Public Health, Cuernavaca, Morelos, Mexico

^24^National Agency for Public Health of the Ministry of Health, Labour and Social Protection of the Republic of Moldova

^25^National Institute for Public Health and the Environment (RIVM), Centre for Sustainability and Environmental Health, Bilthoven, Netherlands

^26^Norwegian institute of Public Health, Oslo, Norway

^27^Department of Hygiene, Graduate School of Medicine, Hokkaido University, Sapporo, Japan

^28^School of Tropical Medicine and Global Health, Nagasaki University, Nagasaki, Japan

^29^Department of Global Health Policy, Graduate School of Medicine, The University of Tokyo, Tokyo, Japan

^30^Department of Environmental Health, Instituto Nacional de Saúde Dr. Ricardo Jorge, Porto, Portugal

^31^EPIUnit - Instituto de Saúde Pública, Universidade do Porto, Porto, Portugal

^32^Laboratório para a Investigação Integrativa e Translacional em Saúde Populacional (ITR), Porto, Portugal

^33^Faculty of Geography, Babes-Bolay University, Cluj-Napoca, Romania

^34^Department of Environmental Health. Rollins School of Public Health, Emory University, Atlanta, USA

^35^Department of Earth Sciences, University of Torino, Italy

^36^Graduate School of Public Health, Seoul National University, Seoul, South Korea

^37^School of Biomedical Convergence Engineering, College of Information and Biomedical Engineering, Pusan National University, Yangsan, South Korea

^38^Institute of Environmental Assessment and Water Research (IDAEA), Spanish Council for Scientific Research (CSIC), Barcelona, Spain

^39^Department of Statistics and Computational Research. Universitat de València, València, Spain

^40^CIBERESP, Madrid. Spain

^41^Department of Public Health and Clinical Medicine, Umeå University, Sweden

^42^Swiss Tropical and Public Health Institute, Allschwill, Switzerland

^43^University of Basel, Basel

^44^Department of Epidemiology and Preventive Medicine, School of Public Health and Preventive Medicine, Monash University, Melbourne, Australia

^45^Climate, Air Quality Research Unit, School of Public Health and Preventive Medicine, Monash University, Melbourne, Australia

^46^Department of Quantitative Methods, School of Medicine, University of the Republic, Montevideo, Uruguay

^47^Department of Environmental Health, Harvard T.H. Chan School of Public Health, Boston, MA, USA

^48^Department of Environmetal Health, Faculty of Public Health, University of Medicine and Pharmacy at Ho Chi Minh City, Ho Chi Minh City, Vietnam

^49^Environment & Health Modelling (EHM) Lab, Department of Public Health Environments and Society, London School of Hygiene & Tropical Medicine, London, UK

^50^School of the Environment, Yale University, New Haven CT, USA

^51^Center for Climate Change Adaptation, National Institute for Environmental Studies, Tsukuba, Japan

^52^Institute of Social and Preventive Medicine (ISPM), University of Bern, Bern, Switzerland

^53^Oeschger Centre for Climate Change Research (OCCR), University of Bern, Bern, Switzerland

^54^Department of Public Health Environments and Society, London School of Hygiene & Tropical Medicine, London, United Kingdom

^55^School of Epidemiology & Public Health, Faculty of Medicine, University of Ottawa, Ottawa, Canada

^56^Environmental Health Science & Research Bureau, Health Canada, Ottawa, Canada

^57^Department of Epidemiology, Instituto Nacional de Saúde Dr. Ricardo Jorge, Lisbon, Portugal

^58^Climate Research Foundation (FIC), Madrid, Spain

^59^Department of Statistics, Computer Science and Applications "G. Parenti", University of Florence, Florence, Italy

^60^National Institute of Environmental Health, Chinese Center for Disease Control and Prevention, Beijing, China

^61^School of Public Health and Social Work, Queensland University of Technology, Brisbane, Australia

**
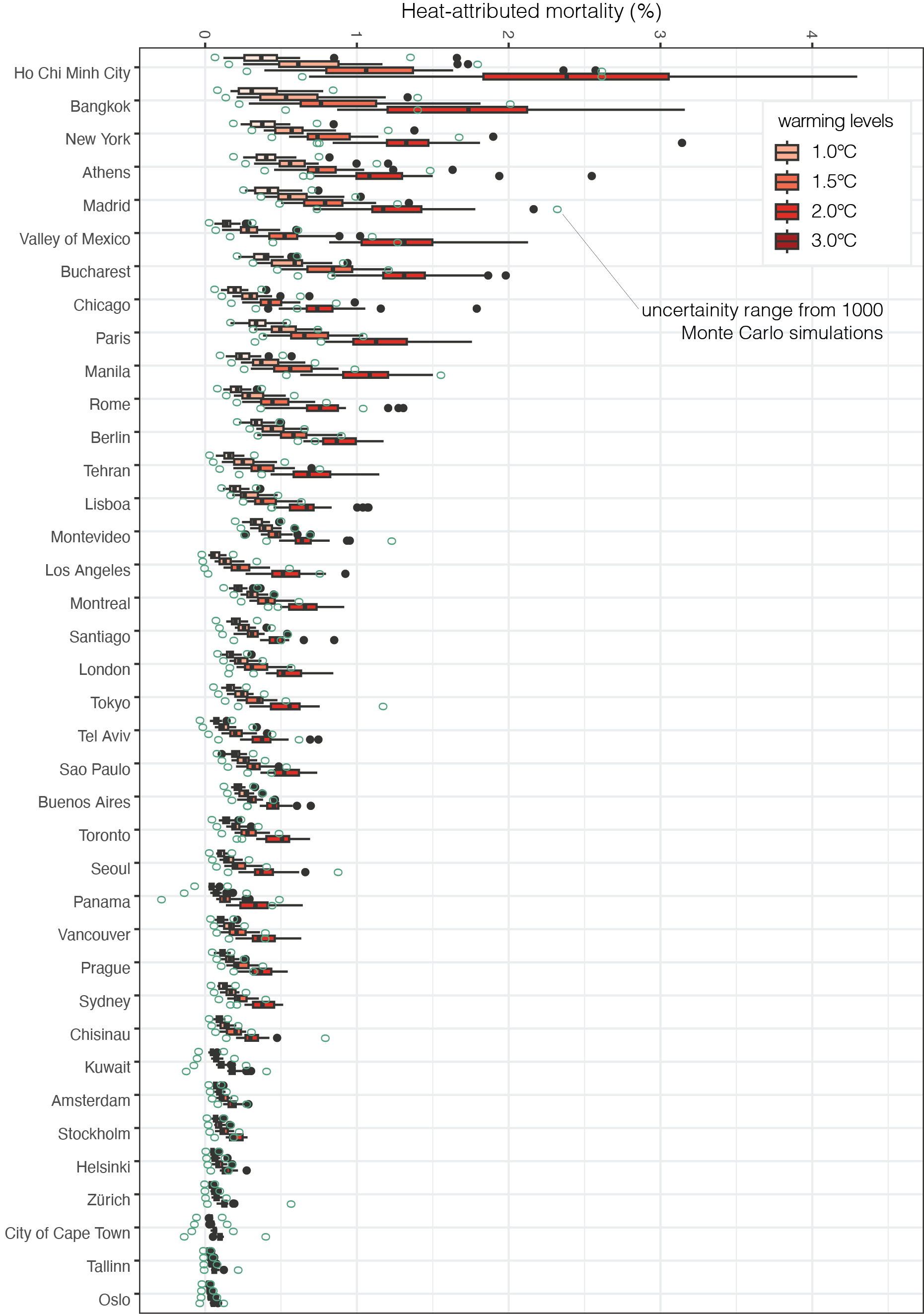
**

**Supplementary Figure 1.** Mortality due to heat for 4 GWLs is shown in boxplots, highlighting the range of 31 CMIP6 GCMs. The 95% confidence intervals (CIs) calculated for the 31 GCMs is indicated by green circles. The values have been obtained by dividing COVID-19-related mortality in 2020/2021 by the average heat-related mortality for each global warming level (GWL), expressed as attributable mortality fraction (%).


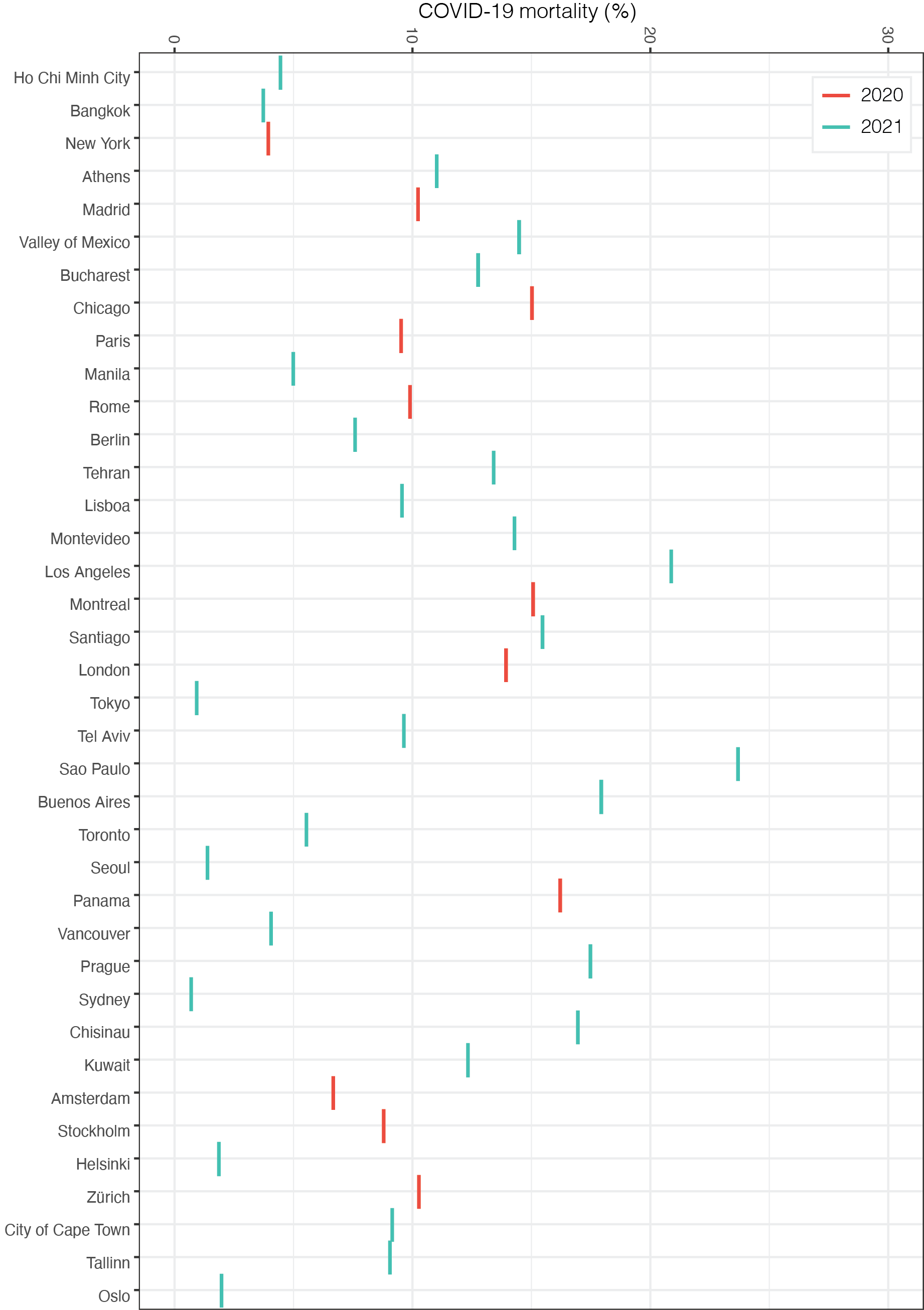


**Supplementary Figure 2.** Maximum mortality due to COVID-19 in either 2020 or 2021.

**Supplementary Table 1.** The number of years required for heat-related mortality to reach COVID-19 mortality in years at four global warming levels. The values represent median of 31 models. 95% confidence intervals are represented in the parenthesis.

| Country | City | +1.0 ºC | +1.5 ºC | +2.0 ºC | +3.0 ºC |
| --- | --- | --- | --- | --- | --- |
| Argentina | Buenos Aires | 84 [55-105] | 67 [48-93] | 60 [39-85] | 40 [26-50] |
| Australia | Sydney | 6 [4-10] | 5 [3-8] | 4 [2-5] | 2 [2-3] |
| Brazil | Sao Paulo | 118 [86-217] | 91 [69-136] | 74 [49-116] | 46 [32-65] |
| Canada | Montreal | 70 [42-97] | 50 [34-65] | 37 [26-52] | 23 [17-31] |
| Canada | Toronto | 41 [26-62] | 29 [18-40] | 20 [13-29] | 11 [8-17] |
| Canada | Vancouver | 40 [20-65] | 25 [18-47] | 20 [11-43] | 12 [7-21] |
| Chile | Santiago | 78 [55-111] | 61 [38-79] | 50 [29-83] | 33 [18-43] |
| Czech Republic | Prague | 156 [104-302] | 107 [66-166] | 83 [50-126] | 48 [32-82] |
| Estonia | Tallinn | 486 [226-767] | 341 [152-611] | 267 [123-537] | 156 [74-234] |
| Finland | Helsinki | 38 [21-65] | 29 [13-52] | 23 [11-42] | 15 [7-20] |
| France | Paris | 29 [18-57] | 19 [12-30] | 15 [9-25] | 9 [5-12] |
| Germany | Berlin | 23 [15-34] | 17 [11-23] | 13 [8-22] | 9 [7-12] |
| Greece | Athens | 28 [14-44] | 20 [9-34] | 15 [7-24] | 10 [4-16] |
| Iran | Tehran | 85 [52-190] | 54 [28-122] | 38 [19-72] | 20 [12-31] |
| Israel | Tel Aviv | 136 [69-304] | 84 [29-150] | 49 [24-90] | 26 [13-42] |
| Italy | Rome | 47 [29-83] | 35 [19-53] | 23 [14-41] | 13 [8-25] |
| Japan | Tokyo | 6 [4-10] | 4 [3-7] | 3 [2-5] | 2 [1-3] |
| Kuwait | Kuwait | 274 [157-590] | 179 [103-307] | 120 [70-173] | 69 [41-85] |
| Mexico | Valley of Mexico | 104 [52-236] | 52 [24-131] | 28 [14-49] | 11 [7-18] |
| Moldova | Chisinau | 184 [128-360] | 126 [83-226] | 85 [63-181] | 57 [36-83] |
| Netherland | Amsterdam | 99 [56-145] | 75 [50-130] | 59 [38-97] | 37 [24-56] |
| Norway | Oslo | 126 [70-188] | 91 [48-185] | 71 [31-137] | 42 [24-67] |
| Panama | Panama | 429 [171-760] | 228 [89-476] | 127 [56-221] | 49 [25-119] |
| Philippines | Manila | 22 [9-37] | 14 [8-21] | 9 [6-17] | 5 [3-8] |
| Portugal | Lisboa | 50 [27-82] | 37 [20-54] | 26 [15-35] | 14 [9-21] |
| Romania | Bucharest | 32 [21-59] | 22 [14-37] | 15 [11-26] | 10 [6-15] |
| South Africa | City of Cape Town | 240 [190-374] | 186 [140-336] | 142 [117-255] | 90 [75-179] |
| South Korea | Seoul | 15 [10-20] | 10 [6-16] | 7 [4-12] | 4 [2-7] |
| Spain | Madrid | 25 [14-39] | 19 [10-26] | 13 [8-21] | 9 [5-14] |
| Sweden | Stockholm | 130 [75-212] | 91 [55-146] | 75 [47-135] | 47 [32-65] |
| Switzerland | Zürich | 283 [189-497] | 191 [123-293] | 144 [86-245] | 86 [54-136] |
| Thailand | Bangkok | 12 [5-23] | 7 [3-18] | 5 [2-13] | 2[ 1-4] |
| UK | London | 83 [46-141] | 62 [38-87] | 45[24-68] | 27 [17-35] |
| Uruguay | Montevideo | 44 [29-59] | 37 [24-48] | 31 [21-54] | 23 [15-30] |
| USA | Chicago | 77 [37-144] | 50 [22-84] | 36 [15-62] | 20 [8-36] |
| USA | Los Angeles | 382 [149-990] | 163 [81-332] | 95 [49-172] | 41 [23-78] |
| USA | NewYork | 11 [5-17] | 7 [3-10] | 5 [2-7] | 3 [1-5] |
| Vietnam | Ho Chi Minh City | 12 [3-37] | 7 [3-18] | 4 [2-12] | 2 [1-7] |

**Supplementary Table 2.** Description of the observed temperature and mortality data in the MCC locations.

| Country | City | Data period | Mortality data origin | Temperature data origin | Notes on data |
| --- | --- | --- | --- | --- | --- |
| Netherland | Amsterdam | 1995 - 2016 | All causes provided by Statistics Netherlands | Mean daily temperature (in ˚C) and relative humidity (%) were obtained from the Royal Dutch Meteorological Institute (KNMI) as 24-hour average based on hourly measurements | Missing data amount for 0.00% and 0.00% of the mortality and temperature series, respectively |
| Greece | Athens | 2001-2010 | All causes provided by Hellenic Statistical  Authority | Mean daily temperature (in ˚C) and relative humidity (%) were computed as the 24-h average based on  hourly measurements  collected from the National observatory of Athens (<http://www.noa.gr/>) from site “Thisio” located in the city of Athens. | Missing data  amount for  0.00% and  7.05% of the  mortality and  temperature  series,  respectively. |
| Thailand | Bangkok | 1999 - 2008 | Non-external (ICD-9: 0-799; ICD-10: A00-R99) mortality, provided the  Ministry of Public  Health, Thailand. | Mean daily temperature (in ˚C) and relative humidity (in %), computed as the  average between daily  minimum and maximum, were obtained from the Meteorological  Department, Ministry of Information and  Communication Technology,  Thailand. | Missing data  amount for  0.00% and  4.99% of the  mortality and  temperature  series,  respectively.  The region  of  Phetchabun  was  excluded  because of  high  percentage  of missing  data. |
| Germany | Berlin | 1993 - 2015 | All causes provided by Research Data Centres of the Federation and the Federal States of  Germany (Forschungsdatenzentr um der Statistischen  Ämter des Bundes und der Länder), | Mean daily temperature (in ˚C), computed as the 24-h average based on hourly measurements, was obtained from the  Climate Data Centre of  the German National  Meteorological Service  (Deutscher Wetterdienst). | Missing data amount for 0.00% and 0.00% of the  mortality and  temperature series,  respectively |
| Romania | Bucharest | 1994 - 2016 | All causes provided by  Romanian National  Institute of Statistics | Meteorological data  (temperature and  relative humidity) were  obtained from stations  operated by the  National Meteorological  Administration of  Romania (NMA RO)  (measurements in  standard climatic terms,  mean daily) by  https://www.ecad.eu/ | Missing data  amount for  0.00% and  0.00% of the  mortality and  temperature  series,  respectively |
| Argentina | Buenos Aires | 2005 - 2015 | Non-external  causes only (ICD-9: 0-  799; ICD-10: A00-R99)  from National Ministry  of Health. | Mean daily temperature (in ˚C) and relative Humidity (in %), computed as the 24-hour average based on hourly  measurements from  one meteorological  station in each city  provided by the  National Weather  Service. | Missing data amount for  0.91% and  0.00% of the mortality and  temperature  series,  respectively |
| USA | Chicago  Los Angeles  New York | 1991 - 2006 | All causes provided by the National Center for Health Statistics (NCHS). | Mean daily temperature  (in ˚C) and relative  humidity (%), computed as the 24-hour average based on hourly measurements, were obtained from the National Climatic Data Center (NCDC) of the National Oceanic and Atmospheric Administration (NOAA). | Missing data  amount for  2.65% and  2.70% of the mortality and temperature series,  respectively. 1 city was  excluded  (Nampa)  because of  high percentage  of missing. |
| Moldova | Chisinau | 2001-2010 | All causes provided by  National Centre for  Health Management. | Mean daily temperature  (in ˚C) computed as the average between daily minimum and  maximum, were  obtained from State  Hydrometeorological  Service, Moldova. A  single weather station  was selected for each  city | Missing data  amount for  0.00% and  0.00% of the  mortality and  temperature series,  respectively |
| South Africa | City of Cape Town | 1997-2013 | All causes provided by  Statistics South Africa | Mean daily temperature (in ˚C) was computed as the average between daily minimum and maximum collected  from the Agricultural  Research Council of  South Africa and the  National Oceanic and  Atmospheric  Administration (NOAA). | Missing data  amount for  0.00% and  12.27% of  the mortality  and  temperature series,  respectively  7 locations  were  excluded  because of a high % of  missing data or unstable  temporal  patterns in  the mortality  data, possibly due  to problems  with data  collection. |
| Finland | Helsinki | 1994 - 2014 | All causes provided by  Statistics Finland | Mean daily temperature (in ˚C), Finnish Meteorological Institute. The weather stations around the country were interpolated onto a 10Å~10 km grid covering the whole of Finland, using a Kriging model. | Missing data  amount for 0.00% and  4.88% of the  mortality and  temperature  series, respectively |
| Vietnam | Ho Chi Minh City | 2010 - 2013 | All causes provided by  Provincial Department  of Health. | Mean daily temperature (in ˚C), and relative humidity (in %) computed as computed from the 24-h average of hourly measurements, were obtained from National Oceanic and Atmospheric Administration's (NOAA) National Climate Data Center (NCDC). A single weather station was selected for each city. | Missing data  amount for  0.00% and  0.57% of the  mortality and  temperature  series,  respectively |
| Kuwait | Kuwait | 2000 - 2016 | Non-external causes  only (ICD-9: 0-799;  ICD-10: A00-R99)  provided by the  National Center for  Health Information,  Ministry of Health,  Kuwait | Mean daily temperature (in ºC) and relative humidity. (in %), computed as the 24-hour average based on hourly measurements from two sources: the Directorate General of  Civil Aviation (Kuwait  Airport) and Kuwait's  Environmental Public  Authority. | Missing data  amount for  0.00% and  0.00% of the  mortality and  temperature  series,  respectively |
| Portugal | Lisboa | 1991 - 2018 | All causes provided by  Statistics Portugal. | Mean daily temperature (in ˚C) was computed as the 24-hour average based on hourly measurements collected from the National Oceanic and Atmospheric Administration (NOAA) | Missing data  amount for  0.00% and  0.00% of the  mortality and  temperature  series,  respectively |
| UK | London | 1991 - 2019 | All causes provided by  the Office of National  Statistics. | Mean daily temperature (in ˚C) and relative humidity (%) were computed as the 24-hour average based on hourly measurements from UKCP09 5kmx5km product | Missing data  amount for  0.00% and  0.00% of the  mortality and  temperature  series,  respectively |
| Spain | Madrid | 1991 - 2014 | Non-external causes  (ICD-9: 0-799; ICD-10:  A00-R99) from the  Spain National Institute  of Statistics. | Mean daily temperature (in ˚C), computed as the 24-hour average based on hourly measurements, and was obtained from  weather stations of the Spain National  Meteorology Agency. A single weather station, located within the urban area or at the near airport, was selected for each city | Missing data  amount for  0.00% and  0.84% of the  mortality and  temperature  series, respectively |
| Philippines | Manila | 2006 - 2019 | All causes provided by  Philippine Statistics  Agency | Mean daily temperature (in ˚C), computed as 24-hour average based on hourly measurements, were obtained from National Oceanic and Atmospheric Administration (NOAA). | Missing data  amount for  0.04% and  0.00% of the  mortality and  temperature  series,  respectively |
| Uruguay | Montevideo | 2012 - 2016 | Non-external causes  are provided by the Ministerio de Salud  Publica (MSP). | Temperature data are  provided by the Instituto Uruguayo de  Meteorología (INUMET) | Missing data  amount for 0.00% and  0.00% of the  mortality and  temperature  series,  respectively |
| Canada | Montreal  Toronto  Vancouver | 1991 - 2015 | All causes collected from Canadian  Mortality Database. | Mean daily temperature (in ˚C) and relative humidity (in %), computed as the 24-hour average based on hourly measurements, were obtained from Environment Canada  collected from  monitoring stations  located closest to the  CMA centre. | Missing data amount for  0.82% and  2.79% of the  mortality and  temperature  series,  respectively |
| Norway | Oslo | 1991 - 2018 | All causes provided by  Norwegian Cause of  Death registry | Mean daily temperature (in ˚C) based on an observational modeled  dataset from the Norwegian Meteorological Institute. | Missing data amount for  2.02% and  3.85% of the  mortality and  temperature  series,  respectively |
| Panama | Panama | 2013 - 2016 | All causes provided by  Instituto Nacional de  Estadística y Censo,  Centro de Información  Estadística. | Temperature data are  provided by the  Empresa de  Transmisión Eléctrica,  S.A. (ETESA). Open  Access. | Missing data  amount for  0.00% and  10.66% of  the mortality  and  temperature  series,  respectively |
| France | Paris | 2000 - 2017 | All causes provided by  French National  Institute of Health and Medical Research  (CepiDC), | Mean daily temperature  (in ˚C), computed as  the mean of the  minimal and maximal  temperature, were  obtained from the  Meteo France. A single  weather station was  selected for each city. | Missing data  amount for  0.25% and  0.04% of the  mortality and  temperature  series,  respectively |
| Czech Republic | Prague | 1994 - 2019 | All causes provided by  the Czech Statistical  Office and the Institute of Health Information  and Statistics | Meteorological data  (temperature and  relative humidity) were obtained from stations operated by the Czech Hydrometeorological  Institute (measurements in  standard climatic terms 7:00, 14:00 and 21:00 local time, and daily means) | Missing data  amount for  0.00% and  0.00% of the  mortality and  temperature  series,  respectively |
| Italy | Rome | 2006 - 2015 | All causes provided by  the obtained from local mortality registries and from the rapid mortality  surveillance system | Mean daily temperature (in ˚C) was computed as the 24-h average based on 6-h measurements  obtained from the Meteorological Service of the Italian Air Force. A single weather station was selected for each city, using the airport monitoring station located closest to the city center. | Missing data  amount for  1.26% and  2.34% of the  mortality and  temperature  series,  respectively.  Data on 12  cities were  initially  collected,  but 1 (Rieti)  was  excluded  because of  potential  problems in  data  collection  (strange  temporal  patterns). |
| Chile | Santiago | 2008 - 2014 | All causes provided by  the Departamento de  Estadísticas e  Información de Salud  (Ministerio de Salud) | Mean daily temperature (in ˚C), computed as 24-hour average based on hourly measurements, were obtained from Sistema de Información Nacional de Calidad del Aire (SINCA), Ministerio del Medio Ambiente. | Missing data  amount for  0.15% and  9.7% of the  mortality and  temperature  series,  respectively |
| Brazil | Sao Paulo | 1997 - 2018 | Non-external  causes only (ICD-9: 0-  799; ICD-10: A00-R99)  from the Ministry of  Health. | Mean daily temperature (in ˚C) and relative humidity (in %), computed from the 24-h average of hourly measurements, from weather stations  located within the urban area provided by National Institute of  Meteorology of Brazil | Missing data  amount for  1.85% and  3.21% of the  mortality and  temperature  series,  respectively |
| South Korea | Seoul | 1997 - 2018 | All causes provided by  Korea Bureau of  Statistics | Mean daily temperature (in ˚C) and relative humidity (in %), computed as the 24- hour average based on hourly measurements,  were obtained from  weather stations  located within the urban area managed by Korea Meteorological Administration. | Missing data  amount for  0.00% and  0.01% of the  mortality and  temperature  series,  respectively |
| Sweden | Stockholm | 1991 - 2016 | All causes provided by  the Swedish Cause of  Death Register at the  Swedish National  Board of Health and  Welfare | Mean daily temperature (in ˚C) and relative humidity (%), computed as the 24-hour average based on hourly measurements, were obtained from the Environment and Health Administration. | Missing data amount for 0.00% and  2.06% of the mortality and  temperature  series,  respectively |
| Australia | Sydney | 1991 - 2009 | Non-external  causes only (ICD-9: 0-  799; ICD-10: A00-R99)  from Australian Bureau  of Statistics. | Mean daily temperature (in ˚C) and relative humidity  (in %), computed as the 24-hour average based on hourly measurements from meteorological stations located within ≤30 km of each city provided by Australian Bureau of Meteorology. | Missing data  amount for  0.18% and  0.00% of the  mortality and  temperature  series,  respectively |
| Estonia | Tallinn | 1997 - 2019 | All causes provided by  Estonian Causes  of Death Registry | Mean daily temperature (in ˚C) and relative humidity (%) were computed as the 24-h average of hourly measurements  collected from Estonian Environment Agency. | Missing data  amount for  0.0% and  0.0% of the  mortality and  temperature  series,  respectively |
| Iran | Tehran | 2004-2013 | All causes provided by  the Ferdows  organization of  Mashhad Municipality | Mean, Max, Min daily  temperature (in ˚C) and relative humidity (in %), computed as the 24-hour average based on hourly measurements collected from IRAN Meteorological  Organization (IRIMO)  (http://www.irimo.ir) | Missing data  amount for  0.00% and  0.00% of the  mortality and  temperature series respectively |
| Israel | Tel Aviv | 1991 - 2019 | All causes provided by the Israeli Central Bureau of Statistics | Israel Meteorological Service (the most representative station for Tel Aviv, selected after consulting with the meteorological service experts) | Missing data amount for  1.45% and  0.38% of the  mortality and  temperature  series,  respectively |
| Japan | Tokyo | 1991 - 2019 | All causes provided by  Ministry of Health,  Labour and Welfare. | Weather station located within the urban area of the capital city (Japan  Meteorology Agency) | Missing data amount for  0.00% and  0.04% of the  mortality and  temperature  series,  respectively |
| Canada | Toronto | 1991 - 2015 | All causes collected from Canadian  Mortality Database. | Mean daily temperature (in ˚C) and relative humidity (in %), computed as the 24-hour average based on hourly measurements, were obtained from Environment Canada  collected from  monitoring stations  located closest to the  CMA centre. | Missing data  amount for  0.82% and  2.79% of the  mortality and  temperature  series,  respectively. |
| Mexico | Valley of Mexico | 1998 - 2014 | All causes provided by  National Institute of  Statistics, Geography  and Informatics | Mean daily temperature  (in ˚C) and relative  humidity (%) were  computed as the 24-  hour average based on  hourly measurements  collected through the  Servicio Meteorológico  Nacional (SMN) and the Instituto Nacional  de Ecología y Cambio  Climático (INECC). | Missing data  amount for  0.00% and  27.03% of  the mortality  and  temperature  series,  respectively |
| Switzerland | Zürich | 1995 - 2013 | Non-external causes  only other than  accidents (ICD-  10codes A00-R99,  V01-V99, W00-X59)  provided from Federal  Office of Statistics  (Switzerland) | Mean daily temperature (in ˚C) and relative humidity (%), computed as the 24-hour average based on hourly measurements, were obtained from the  IDAWEB database (a  service provided by  MeteoSwiss, the Swiss  Federal Office of  Meteorology and  Climatology). A single  weather station located within or near the urban area was selected for each city. | Missing data  amount for  0.0% and  0.0% of the  mortality and  temperature  series,  respectively |

**Supplementary Table 3.** National COVID-19 mortality, total mortality and average mortality fraction in 2020 and 2021. Percentage is represented by % in the table.

| Country name | City | COVID-19 in 2020 | COVID-19 in 2021 | Total mortality in 2020 | Total mortality in 2021 | % in 2020 | % in 2021 | Used COVID-19 % |
| --- | --- | --- | --- | --- | --- | --- | --- | --- |
| Netherland | Amsterdam | 11,432 | 9,492 | 169,182.55 | 171,823.83 | 6.76 | 5.52 | 6.76 |
| Greece | Athens | 4,838 | 15,952 | 130,522.91 | 143,656.48 | 3.71 | 11.10 | 11.10 |
| Thailand | Bangkok | 61 | 21,637 | 521,772.35 | 567,223.94 | 0.01 | 3.81 | 3.81 |
| Germany | Berlin | 33,071 | 78,531 | 989,614.36 | 1,023,311.76 | 3.34 | 7.67 | 7.67 |
| Romania | Bucharest | 15,767 | 42,985 | 298,611.38 | 334,597.90 | 5.28 | 12.85 | 12.85 |
| Argentina | Buenos Aires | 43,245 | 73,924 | 386,110.88 | 410,217.33 | 11.20 | 18.02 | 18.02 |
| USA | Chicago | 7,860 | 4,126 | 52,037.00 | 47,077.00 | 15.10 | 8.76 | 15.10 |
| Moldova | Chisinau | 2,985 | 7,290 | 38,064.45 | 42,784.66 | 7.84 | 17.04 | 17.04 |
| South Africa | City of Cape Town | 28,469 | 62,676 | 554,208.16 | 678,972.26 | 5.14 | 9.23 | 9.23 |
| Finland | Helsinki | 592 | 1,122 | 55,295.43 | 57,626.58 | 1.07 | 1.95 | 1.95 |
| Vietnam | Ho Chi Minh City | 35 | 32,359 | 596,225.74 | 713,173.57 | 0.01 | 4.54 | 4.54 |
| Kuwait | Kuwait | 934 | 1,534 | 14,188.88 | 12,350.83 | 6.58 | 12.42 | 12.42 |
| Portugal | Lisboa | 6,906 | 12,049 | 123,564.97 | 124,934.28 | 5.59 | 9.64 | 9.64 |
| UK | London | 94,998 | 82,399 | 677,520.46 | 650,155.11 | 14.02 | 12.67 | 14.02 |
| USA | Los Angeles | 10,359 | 17,278 | 81,783.00 | 82,411.00 | 12.67 | 20.97 | 20.97 |
| Spain | Madrid | 50,837 | 38,568 | 492,602.81 | 450,449.63 | 10.32 | 8.56 | 10.32 |
| Philippines | Manila | 9,244 | 42,260 | 624,454.98 | 832,579.08 | 1.48 | 5.08 | 5.08 |
| Uruguay | Montevideo | 181 | 5,989 | 32,343.14 | 41,670.17 | 0.56 | 14.37 | 14.37 |
| Canada | Montreal | 8,356 | 3,368 | 55,142.00 | 55,142.00 | 15.15 | 6.11 | 15.15 |
| USA | New York | 3,338 | 1,391 | 82,952.00 | 82,952.00 | 4.02 | 1.68 | 4.02 |
| Norway | Oslo | 436 | 869 | 40,346.06 | 42,184.90 | 1.08 | 2.06 | 2.06 |
| Panama | Panama | 4,022 | 3,406 | 24,679.89 | 26,399.14 | 16.30 | 12.90 | 16.30 |
| France | Paris | 64,267 | 56,934 | 668,953.96 | 657,171.43 | 9.61 | 8.66 | 9.61 |
| Czech Republic | Prague | 11,580 | 24,549 | 129,444.08 | 139,726.77 | 8.95 | 17.57 | 17.57 |
| Italy | Rome | 74,159 | 63,243 | 742,985.64 | 709,316.02 | 9.98 | 8.92 | 9.98 |
| Chile | Santiago | 16,608 | 22,507 | 139,367.57 | 144,756.38 | 11.92 | 15.55 | 15.55 |
| Brazil | Sao Paulo | 19,5072 | 424,262 | 1,581,916.58 | 1,784,480.13 | 12.33 | 23.78 | 23.78 |
| South Korea | Seoul | 917 | 4,708 | 305,833.81 | 320,818.23 | 0.30 | 1.47 | 1.47 |
| Sweden | Stockholm | 8727 | 6,583 | 98,357.70 | 91,659.14 | 8.87 | 7.18 | 8.87 |
| Australia | Sydney | 909 | 1,344 | 161,628.32 | 172,110.13 | 0.56 | 0.78 | 0.78 |
| Estonia | Tallinn | 229 | 1,703 | 15,821.31 | 18,633.05 | 1.45 | 9.14 | 9.14 |
| Iran | Tehran | 55,223 | 76,383 | 485,944.50 | 565,963.13 | 11.36 | 13.50 | 13.50 |
| Israel | Tel Aviv | 3,325 | 4,918 | 48,840.03 | 50,569.38 | 6.81 | 9.73 | 9.73 |
| Japan | Tokyo | 3,492 | 14,900 | 1,401,497.10 | 1,470,474.64 | 0.25 | 1.01 | 1.01 |
| Canada | Toronto | 4,530 | 5,664 | 100,681.00 | 100,681.00 | 4.50 | 5.63 | 5.63 |
| Mexico | Valley of Mexico | 125,807 | 17,3621 | 1,175,564.16 | 1,191,788.53 | 10.70 | 14.57 | 14.57 |
| Canada | Vancouver | 901 | 1522 | 36,759.00 | 36,759.00 | 2.45 | 4.14 | 4.14 |
| Switzerland | Zürich | 7873 | 4344 | 76,015.87 | 71,367.92 | 10.36 | 6.09 | 10.36 |

**Supplementary Table 4.** List of CMIP6 GCMs.

| No | GCM Name | Resolution | Ensemble |
| --- | --- | --- | --- |
| 1 | ACCESS-CM2 | native atmosphere N96 grid (144x192 latxlon);  N96 192 x 144 longitude/latitude 85 levels top level 85 km | r1i1p1f1 |
| 2 | ACCESS-ESM1-5 | native atmosphere N96 grid (145x192 latxlon);  r1.1, N96 192 x 145 longitude/latitude 38 levels top level 39255 m | r1i1p1f1 |
| 3 | AWI-CM-1-1-MR | All grid attributes are set for the native grid;  T127L95 native atmosphere T127 gaussian grid 384 x 192 longitude/latitude  95 levels top level 80 km | r1i1p1f1 |
| 4 | BCC-CSM2-MR | T106 (320 x 160 longitude/latitude) 46 levels  top level 1.46 hPa | r1i1p1f1 |
| 5 | CAMS-CSM1-0 | T106 (320 x 160 longitude/latitude) 31 levels  top level 10 mb | r2i1p1f1 |
| 6 | CESM2-WACCM | native 0.9x1.25 finite volume grid (192x288 latxlon);  0.9x1.25 finite volume grid 288 x 192 longitude/latitude 70 levels top level 4.5e-6 mb | r2i1p1f1 |
| 7 | CESM2 | native 0.9x1.25 finite volume grid (192x288 latxlon); 0.9x1.25 finite volume grid 288 x 192 longitude/latitude 70 levels top level 4.5e-6 mb | r1i1p1f1 |
| 8 | CMCC-CM2-SR5 | native atmosphere regular grid 1-degree 288 x 192 longitude/latitude | r1i1p1f1 |
| 9 | CMCC-ESM2 | native atmosphere regular grid 1deg; 288 x 192 longitude/latitude; 30 levels; top at ~2 hPa | r1i1p1f1 |
| 10 | CNRM-CM6-1-HR | data regridded to a 359 gaussian grid (360x720 latlon) from a native atmosphere T359l reduced gaussian grid | r1i1p1f2 |
| 11 | CNRM-CM6-1 | data regridded to a T127 gaussian grid (128x256 latlon) from a native atmosphere T127l reduced gaussian grid | r1i1p1f2 |
| 12 | CNRM-ESM2-1 | data regridded to a T127 gaussian grid (128x256 latlon) from a native atmosphere T127l reduced gaussian grid; atmos: Arpege 6.3 (T127 Gaussian Reduced with 24572 grid points in total distributed over 128 latitude circles (with 256 grid points per latitude circle between 30degN and 30degS reducing to 20 grid points per latitude circle at 88.9degN and 88.9degS) 91 levels top level 78.4 km) | r1i1p1f2 |
| 13 | CanESM5 | T63L49 native atmosphere, T63 Linear Gaussian Grid 128 x 64 longitude/latitude 49 levels top level 1 hPa | r1i1p1f1 |
| 14 | EC-Earth3-CC | T255L91; IFS cy36r4 (TL255, linearly reduced Gaussian grid equivalent to 512 x 256 longitude/latitude 91 levels top level 0.01 hPa) | r1i1p1f1 |
| 15 | EC-Earth3 | T255L91; IFS cy36r4 (TL255, linearly reduced Gaussian grid equivalent to 512 x 256 longitude/latitude; 91 levels; top level 0.01 hPa) | r1i1p1f1 |
| 16 | FGOALS-g3 | native atmosphere area-weighted latxlon grid (80x180 latxlon); (180 x 90 longitude/latitude 26 levels top level 2.19hPa) | r1i1p1f1 |
| 17 | GFDL-CM4 | Cubed-sphere (c96) – 1-degree nominal horizontal resolution; 360 x 180 longitude/latitude; 33 levels; top level 1 hPa | r1i1p1f1 |
| 18 | GFDL-ESM4 | atmos data regridded from Cubed-sphere (c96) to 180,288 interpolation method: conserve_order2; (Cubed-sphere (c96) - 1 degree nominal horizontal resolution 360 x 180 longitude/latitude 49 levels top level 1 Pa) | r1i1p1f1 |
| 19 | INM-CM4-8 | gs2x1.5 2x1.5; 180 x 120 longitude/latitude; 21 levels; top level sigma = 0.01 | r1i1p1f1 |
| 20 | INM-CM5-0 | gs2x1.5 2x1.5; 180 x 120 longitude/latitude; 73 levels; top level sigma = 0.0002 | r1i1p1f1 |
| 21 | IPSL-CM6A-LR | LMDZ grid NPv6, N96; 144 x 143 longitude/latitude; 79 levels; top level 40000 m | r1i1p1f1 |
| 22 | KIOST-ESM | atmos data regridded from Cubed-sphere (c48) to 94X192; (cubed sphere (C48) 192 x 96 longitude/latitude 32 vertical levels top level 2 hPa) | r1i1p1f1 |
| 23 | MIROC6 | native atmosphere T85 Gaussian grid T85; 256 x 128 longitude/latitude; 81 levels; top level 0.004 hPa | r1i1p1f1 |
| 24 | MIROC-ES2L | native atmosphere T42 Gaussian grid; (T42 128 x 64 longitude/latitude 40 levels top level 3 hPa) | r1i1p1f2 |
| 25 | MPI-ESM1-2-HR | spectral T127; 384 x 192 longitude/latitude; 95 levels; top level 0.01 hPa | r1i1p1f1 |
| 26 | MPI-ESM1-2-LR | spectral T63; 192 x 96 longitude/latitude; 47 levels; top level 0.01 hPa | r1i1p1f1 |
| 27 | MRI-ESM2-0 | native atmosphere TL159 gaussian grid (160x320 lat x lon) TL159; 320 x 160 longitude/latitude; 80 levels; top level 0.01 hPa | r1i1p1f1 |
| 28 | NESM3 | T63; (T63 192 x 96 longitude/latitude 47 levels top level 1 Pa) | r1i1p1f1 |
| 29 | NorESM2-LM | finite-volume grid with 1.9x2.5 degree lat/lon resolution; (2 degree resolution 144 x 96 32 levels top level 3 mb) | r1i1p1f1 |
| 30 | NorESM2-MM | finite-volume grid with 0.9x1.25 degree lat/lon resolution; (1 degree resolution 288 x 192 32 levels top level 3 mb) | r1i1p1f1 |
| 31 | TaiESM1 | finite-volume grid with 0.9x1.25 degree lat/lon resolution; (0.9x1.25 degree 288 x 192 longitude/latitude 30 levels top level ~2 hPa) | r1i1p1f1 |

**Supplementary Table 5.** COVID-19- and heat-related mortalities (% of total mortality) at four global warming levels for 38 cities. 95% confidence intervals are represented in the parenthesis.

| City | COVID-19 | +1.0 ºC | +1.5 ºC | +2.0 ºC | +3.0 ºC |
| --- | --- | --- | --- | --- | --- |
| Buenos Aires | 18.02 | 0.22 [0.13-0.33] | 0.27 [0.16-0.39] | 0.30 [0.19-0.46] | 0.45 [0.29-0.69] |
| Sydney | 0.78 | 0.13 [0.05-0.21] | 0.17 [0.07-0.28] | 0.22 [0.10-0.41] | 0.38 [0.18-0.63] |
| Sao Paulo | 23.78 | 0.20 [0.09-0.33] | 0.26 [0.12-0.40] | 0.32 [0.16-0.55] | 0.52 [0.29-0.84] |
| Montreal | 15.15 | 0.22 [0.13-0.36] | 0.30 [0.20-0.47] | 0.41 [0.25-0.63] | 0.66 [0.43-0.95] |
| Toronto | 5.63 | 0.14 [0.05-0.25] | 0.19 [0.09-0.36] | 0.28 [0.12-0.50] | 0.51 [0.22-0.80] |
| Vancouver | 4.14 | 0.10 [0.05-0.20] | 0.17 [0.07-0.27] | 0.21 [0.09-0.41] | 0.35 [0.17-0.69] |
| Santiago | 15.55 | 0.20 [0.08-0.35] | 0.26 [0.10-0.45] | 0.31[0.12-0.55] | 0.47 [0.20-0.84] |
| Prague | 17.57 | 0.11 [0.06-0.18] | 0.16 [0.09-0.27] | 0.21 [0.12-0.39] | 0.37 [0.20-0.58] |
| Tallinn | 9.14 | 0.02 [0.00-0.05] | 0.03 [0.00-0.06] | 0.03 [0.00-0.09] | 0.06 [0.00-0.13] |
| Helsinki | 1.95 | 0.05 [0.01-0.10] | 0.07 [0.02-0.14] | 0.09 [0.03-0.19] | 0.13 [0.05-0.27] |
| Paris | 9.61 | 0.34 [0.18-0.55] | 0.50 [0.33-0.75] | 0.65 [0.39-1.05] | 1.13 [0.77-1.69] |
| Berlin | 7.67 | 0.33 [0.22-0.50] | 0.44 [0.30-0.66] | 0.58 [0.36-0.91] | 0.87 [0.62-1.24] |
| Athens | 11.10 | 0.40 [0.20-0.76] | 0.56 [0.28-1.14] | 0.74 [0.40-1.49] | 1.08 [0.66-2.33] |
| Tehran | 13.50 | 0.16 [0.04-0.33] | 0.25 [0.06-0.53] | 0.36 [0.11-0.77] | 0.68 [0.23-1.29] |
| Tel Aviv | 9.73 | 0.07 [-0.02-0.19] | 0.12 [-0.01-0.32] | 0.20 [0.03-0.45] | 0.37 [0.10-0.82] |
| Rome | 9.98 | 0.21 [0.09-0.38] | 0.29 [0.15-0.60] | 0.44 [0.22-0.81] | 0.76 [0.38-1.40] |
| Tokyo | 1.01 | 0.16 [0.07-0.28] | 0.25 [0.10-0.40] | 0.35 [0.14-0.54] | 0.56 [0.23-0.89] |
| Kuwait | 12.42 | 0.05 [-0.03-0.13] | 0.07 [-0.04-0.20] | 0.10 [-0.06-0.28] | 0.18 [-0.11-0.46] |
| Valley of Mexico | 14.57 | 0.14 [0.04-0.32] | 0.28 [0.08-0.62] | 0.52 [0.18-1.11] | 1.31 [0.46-2.51] |
| Chisinau | 17.04 | 0.09 [0.04-0.16] | 0.14 [0.05-0.23] | 0.20 [0.08-0.31] | 0.30 [0.15-0.50] |
| Amsterdam | 6.76 | 0.07 [0.03-0.12] | 0.09 [0.04-0.15] | 0.11 [0.06-0.20] | 0.18 [0.09-0.30] |
| Oslo | 2.06 | 0.02 [-0.01-0.05] | 0.02 [-0.01-0.06] | 0.03 [-0.02-0.09] | 0.05 [-0.03-0.13] |
| Panama | 16.30 | 0.04 [-0.06-0.16] | 0.07 [-0.13-0.28] | 0.13 [-0.28-0.50] | 0.33 [-1.05-1.17] |
| Manila | 5.08 | 0.23 [0.11-0.52] | 0.37 [0.18-0.73] | 0.56 [0.27-1.00] | 1.08 [0.54-1.72] |
| Lisboa | 9.64 | 0.19 [0.12-0.35] | 0.26 [0.18-0.49] | 0.37 [0.26-0.64] | 0.67 [0.45-1.11] |
| Bucharest | 12.85 | 0.40 [0.22-0.62] | 0.59 [0.33-0.92] | 0.84 [0.48-1.22] | 1.31 [0.84-1.98] |
| City of Cape Town | 9.23 | 0.04 [-0.05-0.12] | 0.05 [-0.06-0.15] | 0.06 [-0.08-0.20] | 0.10 [-0.13-0.31] |
| Seoul | 1.47 | 0.10 [0.04-0.19] | 0.15 [0.06-0.30] | 0.20 [0.08-0.42] | 0.37 [0.16-0.72] |
| Madrid | 10.32 | 0.42 [0.26-0.71] | 0.55 [0.38-1.00] | 0.79 [0.50-1.28] | 1.17 [0.75-2.06] |
| Stockholm | 8.87 | 0.07 [0.02-0.13] | 0.10 [0.03-0.18] | 0.12 [0.04-0.23] | 0.19 [0.07-0.35] |
| Zürich | 10.36 | 0.04 [0.01-0.07] | 0.05 [0.01-0.11] | 0.07 [0.01-0.15] | 0.12 [0.02-0.24] |
| Bangkok | 3.81 | 0.31 [0.09-0.85] | 0.53 [0.15-1.41] | 0.76 [0.23-2.02] | 1.73 [0.54-3.65] |
| London | 14.02 | 0.17 [0.09-0.29] | 0.23 [0.13-0.39] | 0.31 [0.17-0.58] | 0.52 [0.33-0.90] |
| Montevideo | 14.37 | 0.33 [0.21-0.51] | 0.39 [0.25-0.60] | 0.47 [0.27-0.70] | 0.64 [0.41-1.00] |
| Chicago | 15.10 | 0.19 [0.07-0.38] | 0.30 [0.12-0.64] | 0.42 [0.18-0.87] | 0.74 [0.34-1.56] |
| Los Angeles | 20.97 | 0.05 [-0.01-0.20] | 0.13 [-0.01-0.35] | 0.22 [0.01-0.56] | 0.51 [0.03-1.18] |
| NewYork | 4.02 | 0.38 [0.20-0.75] | 0.57 [0.32-1.22] | 0.74 [0.45-1.68] | 1.32 [0.76-2.82] |
| Ho Chi Minh City | 4.54 | 0.37 [0.07-1.36] | 0.61 [0.17-1.81] | 1.06 [0.29-2.62] | 2.38 [0.65-4.50] |
